# High-risk breast lesions: a combined intratumoral and peritumoral radiomics nomogram model to predict pathologic upgrade and reduce unnecessary surgical excision

**DOI:** 10.3389/fonc.2024.1479565

**Published:** 2024-12-18

**Authors:** Tingting Liao, Yuting Yang, Xiaohui Lin, Rushan Ouyang, Yaohong Deng, Jie Ma

**Affiliations:** ^1^ Department of Radiology, Shenzhen People’s Hospital, The Second Clinical Medical College of Jinan University, Shenzhen, China; ^2^ Department of Radiology, The Eighth Affiliated Hospital, Sun Yat-sen University, Shenzhen, China; ^3^ Department of Research & Development, Yizhun Medical AI Co. Ltd., Beijing, China

**Keywords:** breast, radiomics, magnetic resonance imaging, nomograms, high-risk

## Abstract

**Objective:**

This study aimed to develop a nomogram that combines intratumoral and peritumoral radiomics based on multi-parametric MRI for predicting the postoperative pathological upgrade of high-risk breast lesions and sparing unnecessary surgeries.

**Methods:**

In this retrospective study, 138 patients with high-risk breast lesions (January 1, 2019, to January 1, 2023) were randomly divided into a training set (n=96) and a validation set (n=42) at a 7:3 ratio. The best-performing MRI sequence for intratumoral radiomics was selected to develop individual and combined radiomics scores (Rad-Scores). The best Rad-Score was integrated with independent clinical and radiological risk factors by a nomogram. The diagnostic performance of the nomogram was evaluated using the area under the curve (AUC) of the receiver operating characteristic curve, along with accuracy, specificity, and sensitivity analysis.

**Results:**

The nomogram based on the combined intratumoral and peritumoral Rad-Score of the dynamic contrast-enhanced MRI and clinical-radiological features achieved superior diagnostic efficacy in the training (AUC=0.914) and validation set (AUC=0.867) compared to other models. It also achieved a specificity and accuracy of 85.1% and 82.3% during training and 66.7% and 76.2% during validation.

**Conclusion:**

The nomogram encapsulating the combined intratumoral and peritumoral radiomics demonstrated superior diagnostic efficacy in postoperative pathological upgrades of high-risk breast lesions, enabling clinicians to make more informed decisions about interventions and follow-up strategies.

## Introduction

According to the latest global cancer data released by the World Health Organization in 2024, there were approximately 2.3 million new cases of breast cancer worldwide in 2022, underscoring its significant impact on women’s health (1). The European guidelines for the quality management of breast cancer screening and diagnosis categorize breast lesions into five risk-based types according to pathology: B1 for normal tissue, B2 for benign lesions, B3 for lesions of uncertain malignant potential (high-risk lesions), B4 for suspicious malignancy, and B5 for malignant (2). High-risk lesions, which include atypical ductal hyperplasia, atypia lobular hyperplasia, papillary lesions, complex sclerosing adenopathy, mucinous tumors, and flat epithelial atypia, exhibit clinical and biological heterogeneity. These lesions’ varying malignancy risks necessitate distinct clinical diagnoses, treatment strategies, and follow-up procedures. The management of high-risk lesions has significantly changed over the last few years but still faces great controversy (3). Currently, the initial diagnosis of high-risk lesions mostly relies on core needle biopsy (CNB), which only secures a small specimen and carries a risk of malignancy underestimation. High-risk lesions are generally removed by surgery. Some of them can be upgraded to malignant (B5) due to the discovery of conditions like ductal carcinoma *in situ* and invasive ductal carcinoma during the subsequent surgical pathology. The detection rate of such lesions via CNB ranges from 5% to 9.2% of all biopsied lesions (4, 5).

More high-risk lesions are now being detected due to the popularity of breast cancer screening and the advancement of imaging techniques. However, only a small ratio of high-risk lesions are deemed to have higher levels of malignancy based on surgical pathology, while the rest of them can be managed by disease monitoring through regular follow-ups. This results in a high rate of unnecessary biopsy and surgery for high-risk lesions with a low risk of malignancy. Despite the ongoing investigations on imaging characteristics for risk assessments of breast lesions, there is currently no specific imaging characteristic that can stratify the high-risk lesions based on the risk of malignancy. Malignancy risk prediction could be assisted by applying online predictive models (6, 7). Nevertheless, the suboptimal performance of these models might overestimate or underestimate the risk of subsequent malignancy after diagnosing a high-risk lesion. Early and non-invasive assessments that can predict whether the pathological classification of high-risk lesions will upgrade after surgery would enable clinicians to make more informed decisions regarding surgical and follow-up strategies. This, in turn, could minimize unnecessary procedures, reduce medical costs, alleviate patient pain, and ultimately benefit patients.

Radiomics is an emerging new technique that can transform medical images into high-dimensional and minable quantitative features by leveraging high throughput feature extraction (8). It can provide comprehensive and precise evaluations of lesions based on images. In addition to the characterization within the tumors, more information can be extracted at the peritumor region using radiomics, which has been demonstrated to improve prediction performance on various clinical tasks (9–11). Developing a radiomics nomogram that combines image signatures built by radiomics with other clinical factors is one effective approach for explainable clinical utilization. This study aimed to noninvasively predict pathological upgrading of high-risk breast lesions after surgery by intratumor and peritumor radiomics analysis on multiple MRI sequences. To our knowledge, there is currently no study applying radiomics to further stratify high-risk lesions.

## Materials and method

### Subjects recruitment and data collection

The study received approval from the Ethics Committee of Shenzhen People's Hospital (LL-KY-2021624). Patients with high-risk breast lesions confirmed by CNB at Shenzhen People's Hospital from January 1, 2019, to January 1, 2023, were retrospectively recruited for this study. The lesions included atypical hyperplasia, intraductal papilloma, mucinous tumor, flat epithelial atypical hyperplasia, and sclerosing adenopathy. Clinical data, pathology records, and multi-parametric MRI imaging, which includes T1 weighted imaging (T1WI), T2 weighted imaging (T2WI), diffusion-weighted imaging (DWI), and the second phase of dynamic contrast-enhanced MR imaging (DCE-MRI), were collected. The second phase of DCE-MRI was chosen due to the peak enhancement within the first 2 minutes after the injection of contrast medium, which brings richer information than other phases (12). Inclusion criteria were: (1) complete clinical data and a standardized preoperative breast MRI; (2) no prior CNB before the MRI; (3) CNB confirmation of high-risk breast lesions; and (4) detailed postoperative pathological results or complete follow-up data. Exclusion criteria included: (1) Any MRI sequence of poor-quality affecting image analysis and ROI contouring; (2) lesions without enhancement or indistinguishable due to intense background parenchymal enhancement on DCE-MRI; and (3) prior surgery, endocrine therapy, or neoadjuvant chemotherapy before the MRI. Ultimately, 138 patients were included in the study, comprising 38 with postoperative lesion upgrades and 100 without them. These participants were divided into a training set (n=96) and a test set (n=42) using a 7:3 randomized stratified sampling method.

### MRI image acquisition

All patients underwent bilateral breast DCE-MRI examination using Skyra 3.0T and Avanto 1.5T MR scanners (SIEMENS, Germany) and breast-specific coils. Transverse T1WI, T2WI fat suppression, and DWI (b-value=50, 400, 800 s/mm2) were first performed. The DCE-MRI imaging started with a scout scan, followed by the injection of Gd-DTPA after 30 s. Five consecutive scans were acquired after the contrast agent injection, each lasting 1 min. The scanning parameters were TE=1.7 ms, TR=4.7 ms, scanning field of view 360 mm, spacing=0 mm, layer thickness=1.6 mm, number of layers=72, and acquisition matrix=448 × 372.

### Clinical and radiological feature acquisition

Clinical information included age, family history, menopausal status, clinical manifestations (palpable mass, nipple blood/fluid discharge, pain), and immunohistochemical indicators (ER, PR, HER-2, and Ki-67). Patients’ breast DCE-MRI image characteristics were analyzed by two radiologists with more than five years of experience in breast MRI diagnosis. According to the classification criteria of the Breast Imaging Reporting and Data System (BI-RADS, version 5) (13) of the American College of Radiology, the following radiological features of breast MRI were evaluated and included in this study: Amount of fibroglandular tissue (non-dense: almost entirely fat or scattered fibroglandular tissue, dense: heterogeneous or extreme fibroglandular tissue), background parenchymal enhancement (BPE) (minimal, mild, moderate, and marked), enhancement type (mass enhancement, non-mass enhancement), maximum lesion diameter, lymph node metastasis, and time-intensity curve (TIC) type (persistent, plateau, washout).

### Intratumoral and peritumoral region contouring

**Figure 1:**
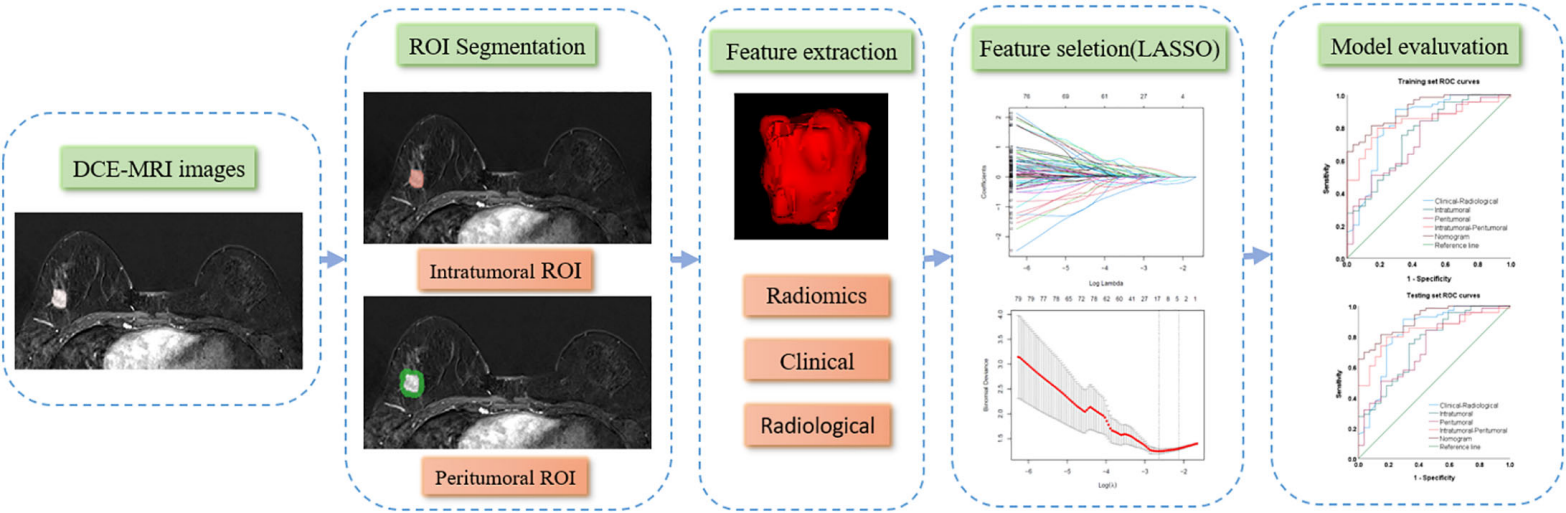
The workflow for processing and analyzing.

T1WI, T2WI, DWI, and DCE-MRI for all patients were retrieved from the PACS at Shenzhen People's Hospital in DICOM format. Breast lesions were manually delineated layer by layer to define the three-dimensional intratumor region of interest (ROI) using the 3D-Slicer software (version 5.2.1, https://www.slicer.org) (14). The peritumoral ROI was established through a 5-mm automatic isotropic expansion (**Figure 1**). The contours were set directly at the enhancement boundaries for lesions exhibiting mass enhancement. For non-mass-enhancing lesions, contours were drawn at the junction between the lesion and normal tissue. After the segmentation of ROI, the repeatability of ROI between two radiologists was evaluated by the intra-class correlation coefficient (ICC) among all the recruited 138 patients.

### Radiomics feature extraction

A comprehensive set of radiomics features was extracted from T1WI, T2WI, DWI, and the second phase of DCE-MRI within the two ROIs (intratumor and peritumor) by the DARWIN, which is a highly flexible platform for imaging research in radiology (15). Images were preprocessed by an isotropic resampling (1×1×1mm) using the B-spline interpolation method to ensure the same image resolution across patients before feature extraction. Both shape, first-order, and texture features were extracted. Texture features were acquired from the gray level co-occurrence matrix (GLCM), gray level dependence matrix (GLDM), gray level run length matrix (GLRLM), gray level size zone matrix (GLSZM), and neighboring gray-tone difference matrix (NGTDM).

### Feature preprocessing and selection

**Figure 2:**
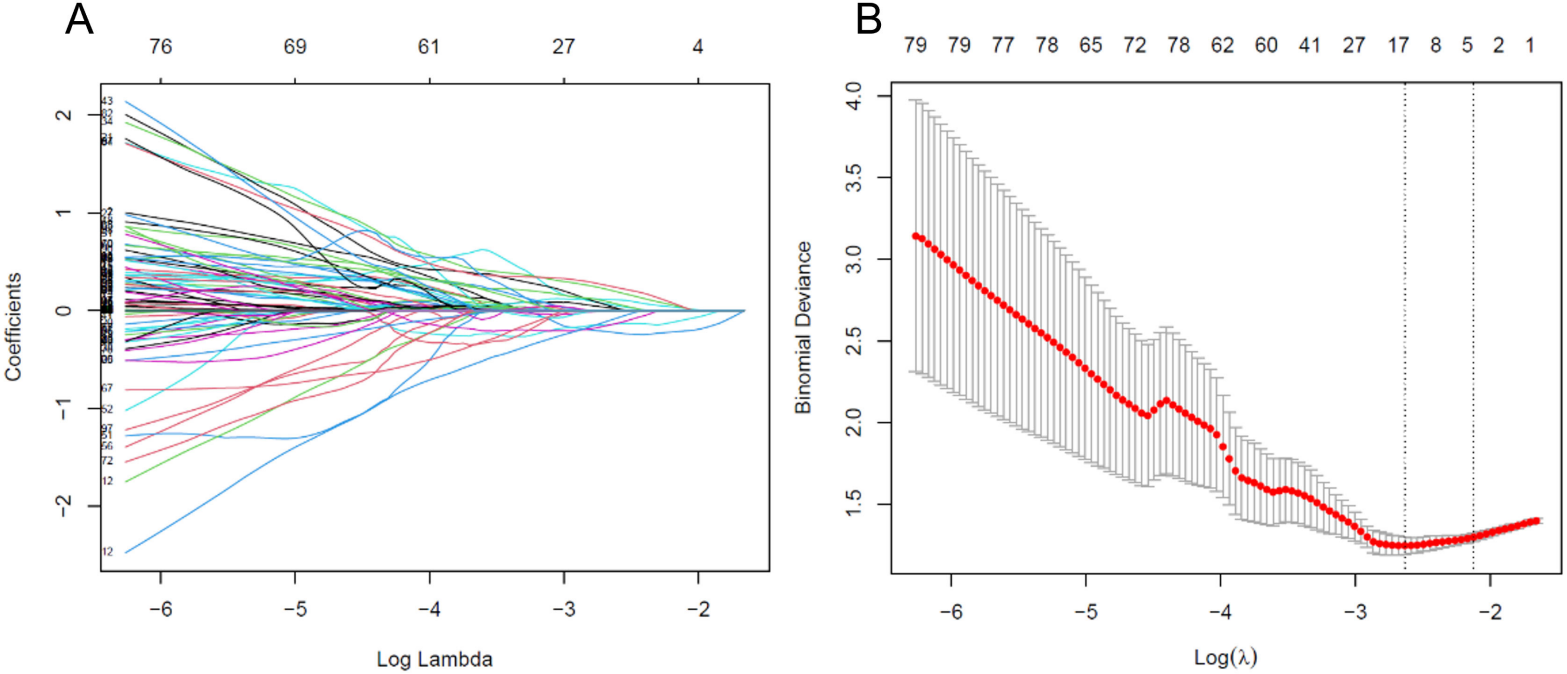
**(A)** Variable trace plot of LASSO regression. The lower x-axis indicates the log λ value, and the upper x-axis indicates the number of features. The y-axis represents the coefficient value of each variable. **(B)** Cross-validation loss plot of LASSO regression.

Feature values were standardized using min-max normalization to scale the data within the range of [-1, 1] across patients. The Least Absolute Shrinkage and Selection Operator (LASSO) regression was employed for dimensionality reduction, identifying the optimal feature set for predicting postoperative pathological upgrading of high-risk breast lesions (**Figure 2**). LASSO reduces regression coefficients towards zero, effectively nullifying many irrelevant features based on the regularization parameter λ was determined using 10-fold cross-validation with criteria set to minimize cross-validation error. Features with non-zero coefficients were retained, and their parameters were used to fit the regression model, forming a radiomics signature.

### Model development and performance evaluation

Intratumoral radiomics models were developed using logistic regression applied to the independently selected radiomics features from the intratumor ROI for all four MRI sequences. The MRI sequence with the best diagnostic efficacy in the training set was selected for further development. The peritumoral radiomics model was developed using the selected radiomics features extracted from the best-performing MRI sequence within the peritumor ROI, and the combined intratumoral-peritumoral model was developed using the merged feature set of the two ROIs. Radiomics scores (Rad-Scores) were calculated for each patient as the outputs from these three radiomics models. A clinical and radiological model was developed using logistic regression on the clinical and radiological features identified as predictive through univariate and multivariate logistic regression analyses.

The discriminative abilities of the developed models were evaluated by the ROC and quantitative metrics, including the AUC, accuracy, sensitivity, specificity, negative predictive value (NPV), and positive predictive value (PPV). Multivariate logistic regression was performed on the best-performing Rad-Score along with predictive clinical and radiological features selected by univariate analysis, and a final nomogram was constructed from the independently predictive factors. The calibration and decision curve analysis (DCA) were performed to assess the nomogram’s calibration and clinical net benefits.

### Statistical analyses

Continuous variables adhering to a normal distribution were presented as mean ± standard deviation and compared using the independent Student’s t-test; discrete variables were reported as frequencies and compared using the χ^2^ test or Fisher’s exact test. The DeLong test was employed to assess any statistical differences in the AUC. A *P*-value of less than 0.05 was deemed significant for all statistical comparisons. During the selection of independent predictive variables, thresholds were set at *P*<0.1 for univariate logistic regression and *P*<0.05 for multivariate logistic regression. All statistical analyses were conducted using SPSS software (version 27.0). Logistic regression analysis and plotting were performed using the “Glm” package in the R language environment (version 4.3.2, https://www.r-project.org/). The “rms” package was utilized for both nomogram development and calibration analysis, while the “rmda” package was used for DCA.

## Results

### Baseline patient characteristics

All 138 included patients were female, aged between 25 and 83 (48.8 ± 12.6) years old. There were 36 cases of atypical hyperplasia, 70 cases of intraductal papilloma, 2 cases of mucinous tumor, 4 cases of flat epithelial atypical hyperplasia, and 26 cases of sclerosing adenopathy. The biopsy methods include core needle biopsy in 93 cases, vacuum-assisted excision in 3 cases, and surgical excision biopsy in 42 cases. One hundred and seventeen cases underwent surgical excision after biopsy, and at least 2 years of imaging follow-ups were available for the 21 cases in patients who did not undergo surgical excision. Thirty-eight patients were found to have ductal carcinoma *in situ* and invasive ductal carcinoma components in the subsequent surgical pathology, with an upgrade rate of 27.5% (38/138). No statistically significant differences in the clinical and radiological characteristics between the training set and the test set were found (*P*>0.05) (**Table 1**).

**Table 1:** Baseline characteristics of the included patients with high-risk breast lesions and their comparisons between the training and test set.

	Training set (n=96)	Test set (n=42)	t/χ2	*P*-value
Age (years old, ± s)	48.53 ± 12.17	49.38 ± 13.54	t=-0.365	0.716
Maximum lesion diameter (mm, $\bar{x}$ ± s)	24.80 ± 14.06	20.81 ± 13.23	t=1.562	0.768
Menopause			χ2 = 0.728	0.393
Yes	37	13		
No	59	29		
Family history			χ2 = 0.028	0.866
Yes	2	0		
No	94	42		
Fibroglandular tissue			χ2 = 1.673	0.196
Non-dense	26	16		
Dense	70	26		
Enhancement type			χ2 = 0.190	0.663
Mass enhancement	51	24		
Non-mass enhancement	45	18		
Palpable mass			χ2 = 0.032	0.857
Yes	26	12		
No	70	30		
Nipple blood/fluid discharge			χ2 = 0.002	0.964
Yes	14	6		
No	82	36		
Pain			–	0.561
Yes	0	3		
No	38	97		
Lymph node metastasis			χ2 = 0.182	0.670
Yes	0	1		
No	96	41		
ER			χ2 = 0.000	1.000
Positive	92	40		
Negative	4	2		
PR			χ2 = 0.616	0.433
Positive	62	30		
Negative	34	12		
HER-2			χ2 = 0.000	1.000
Positive	86	38		
Negative	10	4		
Ki-67(%)			χ2 = 0.405	0.524
≥20	18	6		
<20	78	36		
TIC curve type			χ2 = 3.771	0.152
Persistent	22	10		
plateau	63	22		
Washout	11	10		
BPE			χ2 = 2.289	0.130
Minimal or mild	58	31		
Moderate or marked	38	11		

For fibroglandular tissue: non-dense includes categories a and b, and dense includes categories c and d; $\bar{x}$ ± s: mean ± standard deviation.

### Univariate and multivariate analysis

During univariate logistic regression, the maximum lesion diameter, amount of fibroglandular tissue, Ki-67, and BPE were selected to predict postoperative pathology upgrading (**Table 2**). Three variables, the maximum lesion diameter, Ki-67, and BPE, remained after multivariate logistic regression (**Table 3**) and were used for the clinical-radiological model development.

**Table 2:** Distribution and univariate logistic regression results of the clinical and radiological factors on postoperative pathological upgrading of breast lesions in the training set.

	Upgraded (n=27)	Non-upgraded (n=69)	t/χ2	*P*-value
Age (years old, $\bar{x}$ ± s)	46.96± 11.92	49.14± 12.29	t=-0.79	0.432
Maximum lesion diameter (mm, ± s)	31.15± 15.67	20.29± 10.62	t=3.316	0.002
Menopause			χ2 = 0.553	0.457
Yes	12	25		
No	15	44		
Family history			–	0.077
Yes	2	0		
No	25	69		
Fibroglandular tissue			χ2 = 5.928	0.015
Non-dense	3	25		
Dense	24	44		
Enhancement type			χ2 = 0.374	0.541
Mass enhancement	13	38		
Non-mass enhancement	14	31		
Palpable mass			χ2 = 0.743	0.389
Yes	9	17		
No	18	52		
Nipple blood/fluid discharge			χ2 = 0.079	0.778
Yes	3	11		
No	24	58		
Pain			–	1.000
Yes	0	2		
No	27	67		
Lymph node metastasis			–	1.000
Yes	0	1		
No	27	68		
ER			χ2 = 0.000	1.000
Positive	26	66		
Negative	1	3		
PR			χ2 = 2.859	0.091
Positive	21	41		
Negative	6	28		
HER-2			χ2 = 0.000	1.000
Positive	3	7		
Negative	24	62		
Ki-67 (%)			χ2 = 21.311	<0.001
≥20	13	5		
<20	14	64		
TIC curve type			χ2 = 0.018	0.991
Persistent	6	16		
Plateau	18	45		
Washout	3	8		
BPE			χ2 = 13.609	<0.001
Minimal or mild	10	53		
Moderate or marked	17	16		

For fibroglandular tissue: non-dense includes categories a (almost entirely fat) and b (scattered fibroglandular tissue), and dense includes categories c (heterogeneous fibroglandular tissue) and d (extreme fibroglandular tissue); $\bar{x}$ ± s, mean ± standard deviation; TIC, time-intensity curve.

**Table 3:** Multivariate logistic regression results of the candidate predictive factors for postoperative pathological upgrading of high-risk breast lesions in the training set.

	β	Waldχ^2^	OR (95%CI)	*P*-value
Maximum lesion diameter	-0.074	5.209	0.929 (0.871-0.990)	*0.002
Ki-67	1.580	4.329	4.854 (1.096-21.500)	*0.037
BPE	2.112	5.589	8.265 (1.435-47.603)	*0.018
Combined intratumoral-peritumoral Rad-Score	0.965	8.999	2.625 (1.397-4.931)	*0.003

OR, odds ratio; CI, confidence interval; Rad-score, radiomcis score.

β, regression coefficient.

*, P-value <0.05.

### Model performance evaluation and comparison

**Figure 3:**
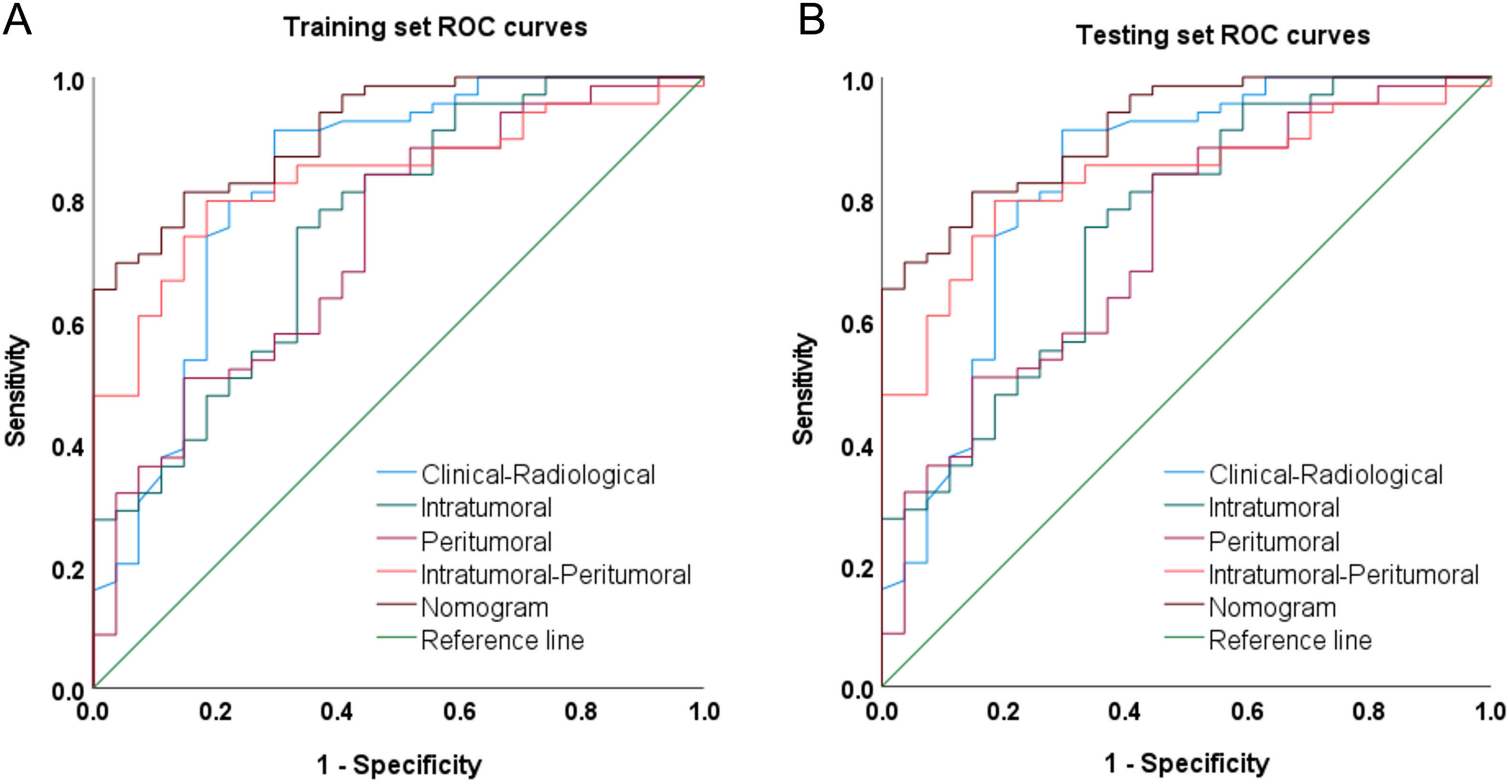
Receiver operating characteristic curves (ROCs) of the clinical-radiological model, intratumoral radiomics model, peritumoral radiomics model, Combined intratumoral-peritumoral radiomics model, and nomogram model on the **(A)** training and **(B)** test set.

The DCE-MRI intratumor radiomics model achieved the best training AUC of 0.755 compared to the rest of MRI sequences (T1WI: 0.649, T2WI: 0.665, DWI: 0.597), as shown in **Table 4**. Therefore, DCE-MRI was selected to construct the peritumoral radiomics and combined models. The combined intratumoral-peritumoral radiomics model displayed superior diagnostic performance compared to the single intratumoral and peritumoral radiomics models. It achieved the highest training AUC of 0.836 and test AUC of 0.768, as detailed in **Table 5** and illustrated by the ROC curves in **Figure 3**. Consequently, the combined Rad-Score and three selected clinical and radiological features were incorporated into the multivariate analysis. Ultimately, the combined intratumoral-peritumoral Rad-Score, maximum lesion diameter, Ki-67, and BPE were identified as independent predictors of postoperative pathological upgrading and were utilized in constructing the nomogram.

**Table 4:** Comparison of training diagnostic efficacy of intratumoral radiomics models built from different MRI sequences.

	AUC (95%CI)	SEN	SPE	ACC	PPV	NPV
T1WI	0.649 (0.538-0.760)	0.491	0.767	0.614	0.722	0.550
T2WI	0.655 (0.560-0.778)	0.547	0.744	0.635	0.725	0.571
DWI	0.597 (0.483-0.712)	0.755	0.442	0.614	0.625	0.594
DCE-MRI	0.755 (0.644-0.865)	0.754	0.667	0.729	0.852	0.514

**Table 5:** Performance of the developed models on predicting the postoperative pathological upgrade in the training and test sets.

	AUC (95%CI)	SEN	SPE	ACC	PPV	NPV
Training						
Clinical-radiological model	0.829 (0.724-0.933)	0.913	0.704	0.854	0.887	0.760
Intratumoral radiomics model	0.755 (0.644-0.865)	0.754	0.667	0.729	0.852	0.514
Peritumoral radiomics model	0.729 (0.616-0.842)	0.841	0.556	0.760	0.829	0.577
intratumoral-peritumoral radiomics model	0.836 (0.755-0.917)	0.797	0.815	0.802	0.917	0.611
Nomogram	0.914 (0.859-0.969)	0.811	0.851	0.823	0.933	0.639
Test						
Clinical-radiological model	0.804 (0.629-0.937)	0.613	0.909	0.690	0.950	0.455
Intratumoral radiomics model	0.765 (0.614-0.917)	0.909	0.581	0.667	0.947	0.435
Peritumoral radiomics model	0.727 (0.549-0.906)	0.818	0.677	0.714	0.913	0.474
intratumoral-peritumoral radiomics model	0.768 (0.628-0.909)	0.645	0.909	0.714	0.952	0.476
Nomogram	0.867 (0.760-0.973)	0.933	0.667	0.762	0.609	0.947

CI, confidence interval; SEN, Sensitivity; SPE, Specificity; ACC, Accuracy; PPV, positive predictive value; NPV, negative predictive value.

**Figure 4:**
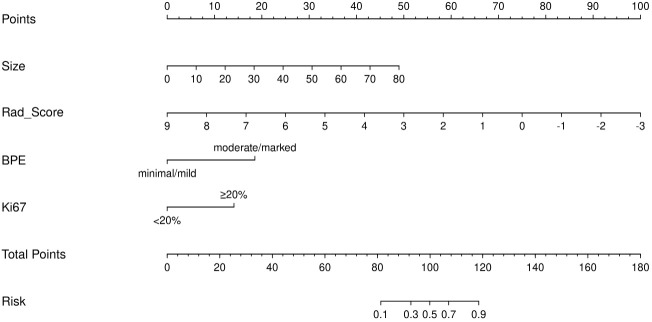
Nomogram composed of the independent predictive factors, including maximum lesion diameter (size), intratumoral-peritumoral Rad-Score, background parenchymal enhancement (BPE), and Ki-67, for the risk of postoperative pathological upgrade.

**Figure 5:**
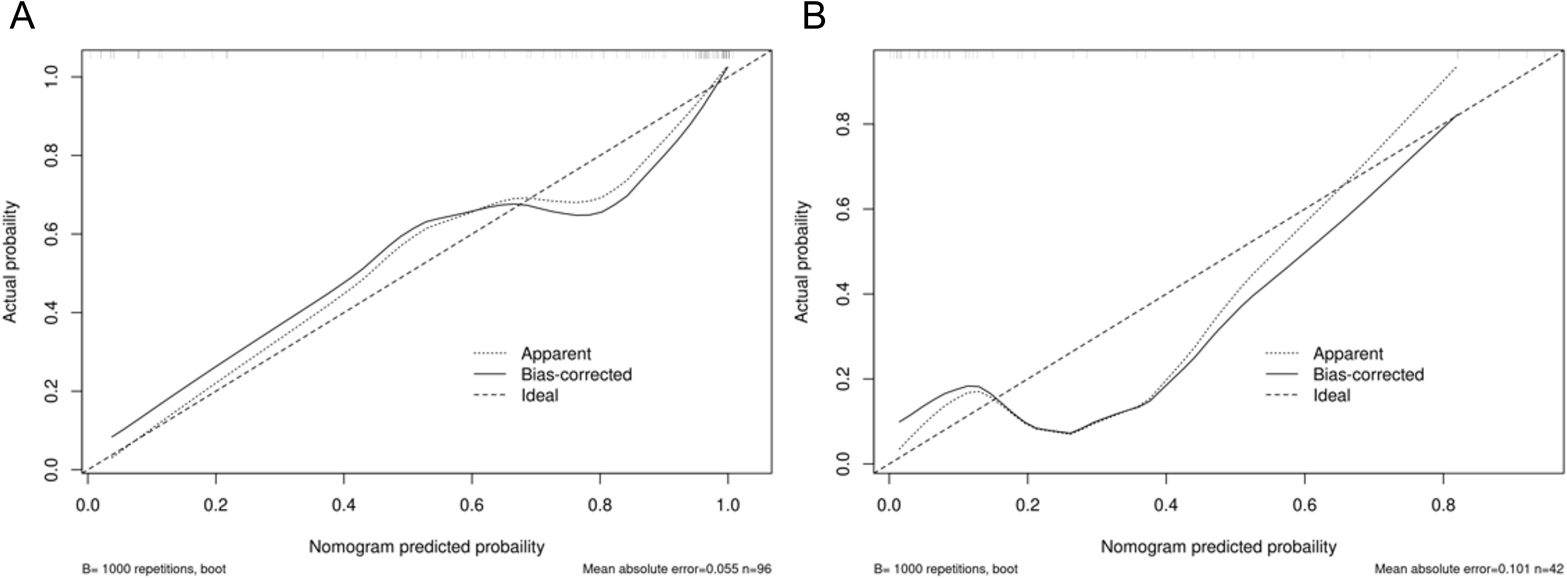
Calibration curves of the final constructed nomogram model in predicting postoperative pathological upgrade in the **(A)** training and **(B)** test set.

**Figure 6:**
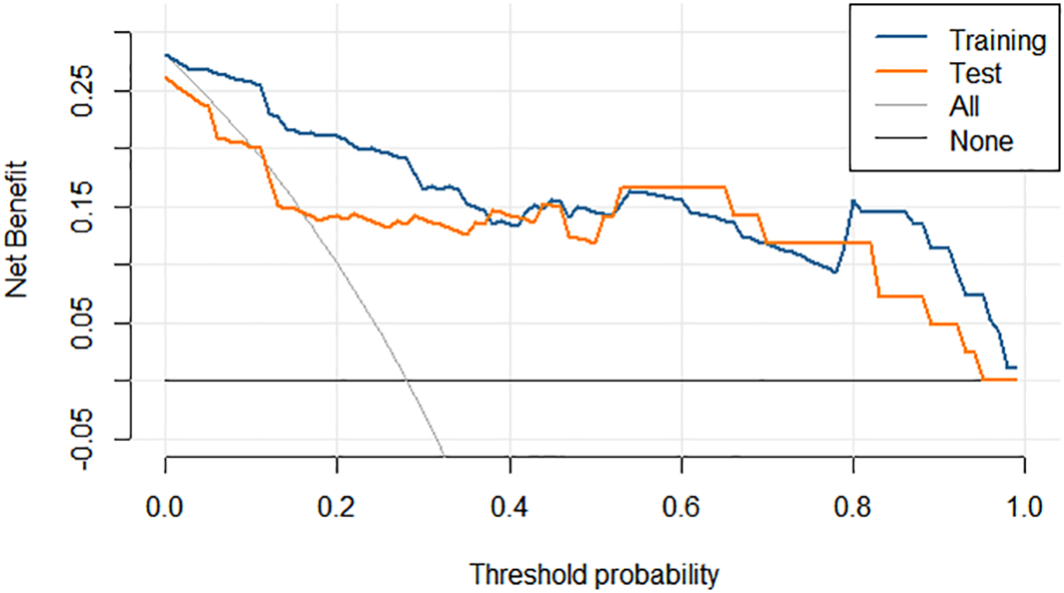
Decision curve analysis of the nomogram in the training and test set.

Integrating the intratumoral-peritumoral Rad-Score with clinical and radiological features, the nomogram demonstrated the highest diagnostic performance. In the training set, the nomogram reached a higher AUC value of 0.914, compared to 0.829 (*P*=0.022) by the clinical and radiological model, 0.755 (*P*=0.009) by the intratumoral radiomics model, 0.729 (*P*=0.006) by the peritumoral radiomics model, and 0.836 (*P*=0.034) by the combined radiomics model. The nomogram also recorded the highest AUC of 0.867 in the test set. Furthermore, the nomogram exhibited superior specificity and accuracy relative to other models, as reported in **Table 5**, **Figure 3**. The visualization of the nomogram is depicted in **Figure 4**. Calibration analysis, shown in **Figure 5**, demonstrated a high consistency between predicted and actual probabilities of the nomogram model in forecasting postoperative pathological upgrading across training and test sets. DCA indicated that the nomogram provided greater clinical benefit than interventions for all patients, with significant net benefits shown in the risk curves for thresholds greater than 0 and 0.15 in both training and test sets, as shown in **Figure 6**.

## Discussion

In recent years, the management of high-risk lesions has sparked debate. Some scholars advocate for surgical excision of high-risk lesions, citing the potential for cancerous tissues beyond the biopsy site and the risk of malignancy upgrade (16). However, many lesions resected surgically are ultimately confirmed as benign in postoperative pathology, prompting proposals for more conservative approaches, such as vacuum-assisted biopsy or excision and observational follow-up, to prevent unnecessary surgeries (17, 18). The non-invasive and precise prediction of postoperative escalation in high-risk lesions using radiomics at the diagnostic stage could aid clinicians in formulating appropriate treatment or surveillance strategies.

Currently, there are few studies on the correlation between breast MRI and the postoperative pathological upgrading of high-risk lesions. Preibsch et al. (19) found that the rate of upgrading of lesions >20 mm was low, whereas Crystal et al. (20) concluded that the size and morphology of the lesions on MRI had no diagnostic value for the rate of upgrading of high-risk lesions. The results of this study showed that the maximum diameter of the lesion was an independent risk factor for the upgrading of high-risk lesions with a positive correlation, which is different from those reported in the literature. This observation could be attributed to the biological characteristics of rapid and infiltrative growth of high-risk breast lesions with subsequent upgrading. You et al. (21) found that high-risk lesions with high degrees of BPE in DCE-MRI had a high rate of pathological upgrade by the univariate and multivariate regression analysis. Our study also suggests that moderate to marked BPE is an independent risk factor for upgrading. Previous studies have confirmed that the degree of BPE is closely related to estrogen level, which changes with the menstrual cycle and is a predictor associated with breast cancer risk (22). Therefore, BPE may suggest a correlation between high-risk breast lesions and the risk of breast cancer development. In addition, our study found that the level of expression of Ki-67 was correlated with the upgrading of high-risk lesions. Previous studies have shown that, as a cell proliferation-associated protein, Ki-67 expression in breast cancer is closely related to the degree of malignancy, invasiveness, and prognosis of the tumor (23). Therefore, the higher the value of Ki-67, the stronger the proliferative activity of tumor cells, and the higher the risk of malignancy and probability of upgrading the high-risk lesions could be.

Although T1WI and T2WI are superior in displaying anatomical structures, they fall short of DCE-MRI in revealing the complex vascular network of tumors and differentiating between benign and malignant tissues (24, 25). Meanwhile, 45.7% (63/138) of the cases in this study exhibited non-mass enhancement (NME); thus, it is difficult to display NME lesions of the breast on T1WI, T2WI and DWI images. DCE-MRI provides in-depth insights into the tumor vasculature and biological characteristics, addressing these limitations of T1WI and T2WI.

No studies have endeavored to predict the postoperative pathological upgrade of high-risk breast lesions using radiomics. Moreover, most existing clinical research utilizing radiomics has focused solely on the tumor interior, neglecting the radiological and biological data from the surrounding tumor area. The peritumor region of breast cancer can hold crucial biological insights that are challenging to capture with traditional diagnostic imaging techniques, such as angiogenesis, peritumor infiltration of lymphatic and blood vessels, and mesenchymal reactions (26). Several studies have shown high diagnostic efficacy from radiomics models constructed using a 5-mm peritumor region (27, 28). Hence, in this study, we used the 5-mm peritumor area to extract radiomics features and develop radiomics models. The clinical and radiological, intratumoral radiomics, peritumoral radiomics, combined intratumoral-peritumoral radiomics, and the radiomics nomogram model were constructed, with the nomogram demonstrating the highest diagnostic efficacy in both the training set (AUC=0.914) and the test set (AUC=0.867). Thus, the nomogram, which combines the breast DCE-MRI intratumor and peritumor radiomics score with clinical and radiological features, exhibits optimal diagnostic efficacy, providing a solid basis for clinical treatment decisions.

This study, however, has several limitations that necessitate further refinement. Firstly, as a single-center retrospective study, it may exhibit selection bias, potentially reducing the stability and generalizability of the model in other clinical settings. Secondly, this study requires that enrolled cases possess both biopsy and postoperative pathological results, as well as comprehensive MRI imagery and clinical documentation. This rigorous criterion inevitably resulted in a limited data set. Future research endeavors necessitate the ongoing accumulation of an external validation cohort to substantiate the model’s efficacy. Thirdly, using a 5-mm expansion for the peritumor ROI means that some potentially informative peritumor tissue beyond this margin was excluded from the radiomics feature extraction, which could further enhance the predictive performance for the pathological upgrade.

## Conclusion

The radiomics nomogram model developed from intratumoral and peritumoral DCE-MRI radiomics, combined with clinical and radiological features, has demonstrated high diagnostic performance for predicting the postoperative pathological upgrade of high-risk breast lesions. This capability to stratify risk, particularly in forecasting whether high-risk lesions will upgrade upon surgical resection, aids clinicians in making personalized clinical decisions for patients with high-risk lesions. It helps select the most beneficial treatment or follow-up regimens, reducing healthcare costs and unnecessary surgeries.

## Data Availability

The datasets presented in this article are not readily available because the datasets generated and analyzed during the current study are available from the corresponding author upon reasonable request. Requests to access the datasets should be directed to cjr.majie@vip.163.com.
